# Active Edible Coatings to Mitigate Postharvest Diseases Causing Waste of Blueberries, Strawberries, and Cherry Tomatoes

**DOI:** 10.3390/foods15010011

**Published:** 2025-12-19

**Authors:** Mara Pasqualicchio, Chahinez Hadjila, Ornella Incerti, Maria Maddalena Cavalluzzi, Giovanni Lentini, Giuseppe Celano, Maria De Angelis, Antonio Ippolito, Simona Marianna Sanzani

**Affiliations:** 1 Department of Soil, Plant and Food Sciences, University of Bari Aldo Moro, via Amendola 165/a, 70126 Bari, Italy; m.pasqualicchio3@phd.uniba.it (M.P.); hadjilachahinez2608@gmail.com (C.H.); ornella.incerti@uniba.it (O.I.); giuseppe.celano@uniba.it (G.C.); maria.deangelis@uniba.it (M.D.A.); antonio.ippolito@uniba.it (A.I.); 2 Department of Pharmacy-Pharmaceutical Sciences, University of Bari Aldo Moro, via Orabona 4, 70125 Bari, Italy; mariamaddalena.cavalluzzi@uniba.it (M.M.C.); giovanni.lentini@uniba.it (G.L.)

**Keywords:** active packaging, phytopathogens, storage, fungal rots, GRAS compounds, *Moringa oleifera*

## Abstract

Packaging can help prolong the shelf life of perishable agrifoods. In the present investigation, edible coatings were tested to reduce food waste caused by filamentous fungi and increase the shelf-life of high-value products such as strawberries, tomatoes, and blueberries. Different combinations of sodium alginate and calcium chloride, and various immersion times were tested on tomato as a model. The ability to activate edible coatings with food-grade compounds/extracts, such as sodium bicarbonate or *Moringa oleifera* extract (MLE), was explored. The extract was also tested in vitro against some of the main postharvest pathogens, such as *Botrytis cinerea*, *Alternaria alternata*, *Rhizopus stolonifer*, *Colletotrichum acutatum*, and *Penicillium expansum.* The most suitable composition for the edible coating proved to be 2% sodium alginate and 2% calcium chloride. MLE proved not to reduce fungal growth, except for *A. alternata* and *C. acutatum*. Concerning active coatings, particularly those containing MLE, there was a reduction in the incidence of rots on strawberries (−45%) and tomatoes (−59%) as compared to the uncoated control. Furthermore, a reduction in the severity of rots was recorded in all tested fruits (−73% in tomato, −88% in strawberries, −47% in blueberries) as compared to the uncoated control. The active edible coatings could play a role in reducing rots, contributing to the extension of the shelf-life of the selected products.

## 1. Introduction

One of the most important food-related concerns in industrialised countries is food waste during marketing and at consumers’ sites. Main contributing factors include physiological alterations, inadequate storage/transport at all stages, including households, and microbial infection/contamination causing spoilage and food-borne diseases, including mycotoxicosis [1]. The use of active packaging might help mitigate fruit and vegetable waste during storage, distribution, and consumption. Food packaging serves three main functions: (i) protection; (ii) communication; (iii) convenience [2]. It might serve as a barrier to oxygen, moisture, and light, offering protection for the most sensitive foods. 

However, conventional packaging materials cannot actively control senescence and deterioration, which are major causes of waste. Developments in materials science and engineering have led to novel packaging solutions, commonly known as active packaging, that assist in prolonging shelf life and enhancing food safety [3]. Active packaging can be classified into (i) non-migratory active packaging, which mainly refers to scavengers designed to remove unwanted components from the environment inside the packaging, and (ii) active releasing packaging, which primarily relates to emitters that allow controlled migration of desired substances into the packaging environment [4]; in the latter, active ingredients with fungicidal or fungistatic activity might be added to the packaging materials to prevent spoilage by phytopathogens. Conventional packaging is mostly made of polyethylene plastic, which is cost-effective but environmentally unfriendly and contrary to the principle of carbon neutrality [5]. Therefore, the selection of safe and degradable biopolymer packaging solutions has become a priority.

Edible films and coatings are alternative packaging technologies that can meet market standards, being safe for human and animal consumption and for the environment, as they are biodegradable. They might be particularly useful during the secondary shelf-life when the packaging has been opened. Among gel-forming substances in edible coatings, there are hydrocolloids, such as alginate (alginic acid sodium salt), which is a widely used and naturally abundant polysaccharide. Food-grade sodium alginate (E401) is classified as “Generally Recognized as Safe” (GRAS) by the United States Food and Drug Administration (FDA) and is employed in several food applications [6]. In Europe, alginic acid and its salts are included in the European Commission’s list of approved additives [7]. Alginate can form strong gels or insoluble polymers through cross-linking with Ca^2+^ by post-treatment of a CaCl_2_ solution [8]. Several reports have been published on the use of alginate-calcium chloride coatings [9,10]; however, limited information is available on their effectiveness against postharvest rots caused by phytopathogens.

The aim of this investigation was to develop active edible coatings to extend the shelf-life of perishable high-value commodities, such as cherry tomatoes, strawberries, and blueberries, by reducing product wastes caused by relevant filamentous fungi. Strawberry and blueberry fruit have a very short shelf-life because of their susceptibility to mechanical injury, excessive texture softening, physiological disorders, and latent infections caused by several phytopathogens [11]. Their shelf-life in cold storage (0–5 °C) is usually around 2 weeks, followed by 3–4 days at room temperature (~20 °C) [12]. Because of their high perishability, small fruits in conventional agriculture should undergo extensive treatments, which carry the risk of pesticide residue accumulation. Even cherry tomatoes are highly important in the Mediterranean diet due to their versatility, convenience, and nutritional value (vitamins, antioxidants like lycopene, etc.). Agriculturally, cherry tomatoes are favoured because of their fast growth, high yield, and resistance to stresses [13]. Their adaptability across various growing conditions (including greenhouses) points out their enduring popularity and importance. The main fungi responsible for postharvest rots of the above-mentioned commodities are *Alternaria alternata*, *Botrytis cinerea*, *Colletotrichum acutatum*, *Penicillium expansum*, and *Rhizopus stolonifer*, which are responsible for significant economic losses. Furthermore, *A. alternata* and *P. expansum* are recognised as mycotoxin producers, thus compromising product safety [14].

In a previous investigation [15], a series of inorganic salts was tested as active ingredients of alternative packaging. Among them, sodium bicarbonate, classified as a food additive by the European Food Safety Authority (EFSA) and as GRAS by the FDA, emerged as effective in reducing rot of cherry tomatoes and strawberries [15]. Being exempt from residue tolerances on agricultural commodities, it could be applied after harvest as an aqueous solution, vapour, or coating treatment [16]. Moreover, it is readily available, inexpensive, and free from phytotoxicity at low concentrations (<4%) [17]. Another relevant source of safe bioactive compounds is plant extracts. In a previous investigation [18], it was demonstrated that leaf aqueous extracts of *Moringa oleifera* were able to ameliorate the nutritional profile and the storability when used as a biostimulant treatment on lettuce. *M. oleifera* is the most widely cultivated among the 13 species of the *Moringaceae* family, and it has an exceptionally wide variety of uses, including the consumption of its fresh and dry leaves in all the cultivating countries (mainly India, Africa, and the Philippines). Indeed, the leaves are reported to contain substantial amounts of vitamins, phenolics, proteins, and minerals, such as calcium, potassium, magnesium, iron, manganese, and copper [19]. These nutrients are known to scavenge free radicals [20].

In the present investigation, sodium bicarbonate and a *Moringa* leaf aqueous extract were used as active ingredients in edible coatings with the aim of prolonging the shelf-life of perishable commodities, such as cherry tomatoes, strawberries, and blueberries.

## 2. Materials and Methods

### 2.1. Reagents, Plant Materials, and Phytopathogens

Cherry tomatoes (*Solanum lycopersicum* var. *cerasiformae*), strawberries (*Fragaria × ananassa* var. *candonga*), and blueberries (*Vaccinium angustifolium*), without alterations and uniform in size, colour, and ripeness, were purchased from a certified organic farm.

The edible coating was prepared using sodium alginate (NaC_6_H_7_O_6_, SaporePuro, Gioia Group S.r.l., Torino, Italy) and calcium chloride (CaCl_2_, Bindly, BDF Natural Ingredients SL, Gerona, Spain), both of food grade.

The active ingredients were food-grade sodium bicarbonate (NaHCO_3_, CRASTAN S.p.A, Pondera, PI, Italy) and a horseradish tree (*M. oleifera*) extract (MLE). MLE was obtained from *M. oleifera* leaves of plants cultivated in the greenhouses of Impresa Moringa Salento s.r.l. (Nociglia, Lecce, Italy). The dried leaves underwent microwave-assisted extraction, following Admane et al. [18]. Briefly, in a microwave tube, 200 mg of leaf powder was suspended in 2 mL of bi-distilled water and extracted for 5 min at 80 °C. After filtration by Whatman 1 filter paper (Merk, Milan, Italy), the sample was centrifuged for 10 min at 8000× *g*, and the supernatant was lyophilized at −50 °C (LIO25 FP Freeze Dryer, 5Pascal, Trezzano sul Naviglio, MI, Italy) and resuspended in 500 mL of sterile bi-distilled water.

The fungal strains used in the study, namely *Alternaria alternata* A25, *Botrytis cinerea* BC28, *Colletotrichum acutatum* FV68, *Penicillium expansum* Pex04, and *Rhizopus stolonifer* RZ01, came from the Fungal Collection of the Department of Soil, Plant and Food Sciences, University of Bari Aldo Moro, Bari, Italy.

All experiments were performed at least twice.

### 2.2. Selection of Edible Coating Composition

Cherry tomatoes were selected as model fruits because of their physical and physiological characteristics. To optimise coating composition, various concentrations of sodium alginate (1–2%) and calcium chloride (2–4%) were combined; furthermore, various immersion times (3–6 min) of fruits in the cross-linker calcium chloride were used (Table 1). The sodium alginate was dissolved overnight in sterile distilled water on a magnetic stirrer (M2-A, ArgoLAB, Carpi, MO, Italy) and finally homogenised by a commercial blender to remove bubbles.

The fruits were first dipped in the sodium alginate solution for 3 min and then in the calcium chloride solution, according to Table 1. Then they were left to dry on a grid at room temperature. Once dry, they were placed inside plastic containers with an absorbent pad (six fruit per box, three replicates per treatment), packed in a microperforated polyethylene bag to maintain a high relative humidity (80–85%) and stored in the dark at 16 ± 1 °C. The fruits were inspected at 5, 7, 12, and 14 days of incubation by evaluating the incidence (infected fruits, %) and severity (infected fruit surface, %) of rots caused by natural infections. Disease severity was calculated using ImageJ 1.54g software v. Java 1.8.0_345 (64-bit) (https://imagej.nih.gov/ij/download.html (accessed on 1 October 2025)).

### 2.3. Selection of the Active Coating Composition to Be Used on Fruits

Based on previous tests in Section 2.2 (Supplementary Figure S1), to assess the best conditions of sodium alginate-sodium bicarbonate interaction, the active ingredient sodium bicarbonate at 1% concentration, selected according to Incerti et al. [15], was added to the sodium alginate at 1–2% concentrations during the preparation of the coating (Table 2). The duration of the immersion in calcium chloride was scheduled according to Section 2.2. As reported above, cherry tomatoes were used as model fruits.

Sodium bicarbonate was first solubilised in sterile distilled water and then added to the sodium alginate and kept under stirring, making the solution active (active coating). A portion of the sodium alginate was prepared without sodium bicarbonate (non-active coating) to serve as a comparison. Uncoated fruits were used as a control. The fruits were then dipped first in active/non-active sodium alginate and then in calcium chloride. They were left to dry on a grid at room temperature.

Subsequently, fruits were placed in plastic containers with an absorbent pad (six fruit per box, three replicates per treatment), packed with a microperforated polyethylene bag to maintain a high relative humidity (80–85%), and stored at 16 ± 1 °C. Incidence and severity of the disease were evaluated, as reported in Section 2.2, after 7 days of incubation.

### 2.4. Assessment of the Moringa oleifera Extract (MLE) on the Growth of Relevant Fungi

The effect of the MLE was evaluated on the colony growth of relevant fungi for the crops of interest, namely *B. cinerea*, *A. alternata*, *R. stolonifer*, *C. acutatum*, and *P. expansum*. Stock solution of the extract prepared as reported in Section 2.1 was added to molten (50 °C) Potato Dextrose Agar (PDA) (Liofilchem s.r.l., Roseto degli Abruzzi, TE, Italy) before pouring into 90 mm Petri dishes to achieve a 1.5% final concentration. The concentration was selected according to previous trials [18]. PDA dishes without MLE served as a control. The dishes were seeded in the centre with a 5 mm plug of mycelium taken from the edge of an actively growing colony of the selected phytopathogens and incubated at 20 ± 1 °C. Colony diameter (mm) was measured at 4 and 8 days of incubation.

### 2.5. Application of Active Coatings on Fruits to Affect Product Rots

Following the conditions set up in Section 2.3, the active coatings were prepared by amending 2% sodium alginate with sodium bicarbonate 1% or MLE 1.5%. The fruits (tomatoes, strawberries, and blueberries) were first dipped in active/non-active sodium alginate and then in 2% calcium chloride for 3 min. Before coating, to simulate a higher inoculum pressure, fruits were wounded twice (1 mm × 2 mm) in the equatorial zone and immersed for 2 min in a fungal suspension of *A. alternata* (tomatoes, Supplementary Figure S1) or *B. cinerea* (blueberries), with a concentration of 10^4^ conidia/mL, and allowed to dry at room temperature for 30 min. For strawberries, given the high number of latent infections, no artificial inoculation was performed. Once dry, fruits were placed in plastic containers with an absorbent pad (6 replicates of 3 fruit each for strawberries, 3 replicates of 8 fruit each for tomatoes, 3 replicates of 25 fruits each for blueberries), packed with a microperforated polyethylene bag to maintain a high relative humidity (80–85%), and stored at the suitable temperature for the different fruit types: 5 ± 1 °C (strawberries), 16 ± 1 °C (tomatoes and blueberries) for evaluating the product shelf-life. The incidence and the severity of the disease were evaluated, as reported in Section 2.2, after 12 days of incubation.

### 2.6. Statistical Analyses

The effect of treatments was expressed by a control index (CI) using the following formula: CI (%) = [(C − T)/C] × 100, where C and T represent the mean value of each parameter assessed in control and treated samples, respectively.

All data were subjected to a one-way analysis of variance (One-way ANOVA) using the statistical software package Statistics for Windows v7.0.61.0 (StatSoft, Tulsa, OK, USA). Means were separated utilising Fisher’s test as a post hoc test, performed at a *p* < 0.05 significance level. Percentage data were subjected to arcsine square root transformation before ANOVA analysis.

## 3. Results and Discussion

### 3.1. Effect of Edible Coating Composition on the Shelf-Life of Tomatoes

The shelf-life of tomatoes as model fruits was evaluated after applying an edible coating based on sodium alginate and calcium chloride. Specifically, the assay concerned the use of different concentrations of sodium alginate (1, 1.5, and 2%) in which the fruits were dipped for 3 min, and of calcium chloride (2 and 4%) as a cross-linker in which they were dipped for 3 or 6 min.

Sodium alginate concentration strongly influences the viscosity, thickness, mechanical strength, and barrier properties of the resulting edible coating, with higher concentrations forming denser films that may better limit moisture and gas exchange but can become excessively thick or brittle if overconcentrated. Calcium chloride, used as a cross-linking agent, further modifies coating performance by strengthening the alginate network, enhancing structural integrity, and improving moisture barrier efficiency. However, excessively high levels can cause over-hardening, surface whitening, and reduced flexibility. Therefore, the functional properties of alginate-based edible coatings depend on achieving a balanced ratio between alginate and calcium ions to produce a uniform, stable, and effective film.

**Figure 1:**
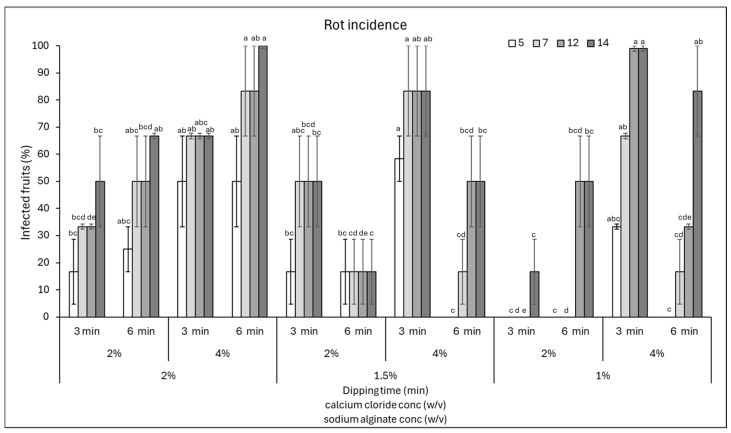
Disease incidence (infected fruits, %) at 5, 7, 12, and 14 days of storage on cherry tomatoes coated with various combinations of sodium alginate (1–2%)—calcium chloride (2 and 4%) concentrations and immersion times in calcium chloride (3 and 6 min). Data are the average of three replicates ± standard error of the mean (SEM). For each time point, bars with different letters are significantly different (*p* < 0.05).

The fruits were stored for 14 days and inspected periodically for the occurrence of rots caused by natural infections. The data showed that the lowest incidence of rots on tomatoes was observed when a coating of 1% sodium alginate was soaked in 2% calcium chloride for 3 min, since the rots did not develop until the end of the incubation (14 days) (Figure 1). In addition, a 17% incidence was found when using 1.5% sodium alginate and 2% calcium chloride for 6 min for all 14 days of incubation. Finally, in the presence of 2% sodium alginate and immersion in 2% calcium chloride for 3 min, 17% of the fruit area was infected at 5 days of incubation.

**Figure 2:**
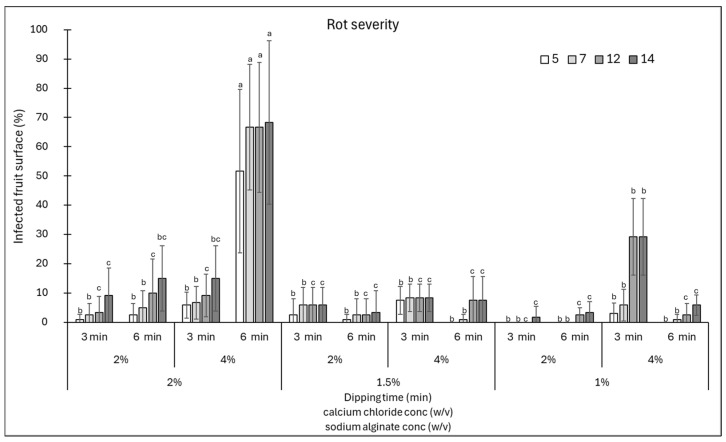
Disease severity (area of infected fruit, %) at 5, 7, 12, and 14 days of storage on cherry tomatoes coated with various combinations of sodium alginate (1–2%)—calcium chloride (2 and 4%) concentrations and immersion times in calcium chloride (3 and 6 min). Data are the average of three replicates ± standard error of the mean (SEM). For each time point, bars with different letters are significantly different (*p* < 0.05).

Similarly, the response of the fruits in terms of disease severity was evaluated by estimating the area of fruit affected by rots (Figure 2). Almost all combinations of sodium alginate and calcium chloride, except for the combination sodium alginate 2% + calcium chloride 4% for 6 min and sodium alginate 1% + calcium chloride 4% for 3 min, showed a disease severity below 20% (Figure 2). Therefore, from the data obtained, it is possible to conclude that a longer shelf-life of fruits might be obtained with coatings made of 1% sodium alginate + 3 min immersion in 2% calcium chloride. In addition, good results were obtained by varying the sodium alginate to 2 or 1.5%, in the latter case extending the immersion in calcium chloride to 6 min. Prolonged immersion in calcium chloride resulted in a higher incidence and severity of rots, likely because the increased coating thickness created humid microenvironments that favoured the proliferation of microorganisms on the fruit surface. Indeed, Cisneros-Zevallos and Krochta [21] reported that oxygen and carbon dioxide concentrations in the micro-gap between the coating and the fruit surface might vary according to the amount of dry coating load or film thickness. Thus, the coating solution properties and compositions could modify internal gases, consequently affecting the risk of infection.

### 3.2. Effect of the Composition of the Active Edible Coating in Reducing Rots

**Figure 3:**
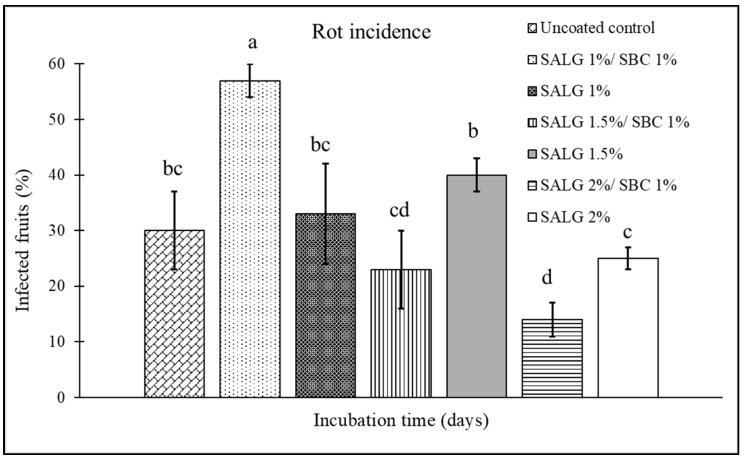
Disease incidence (infected fruits, %) on cherry tomatoes at 7 days of storage for the different treatments: control (uncoated); active coating with 1% sodium alginate and 1% sodium bicarbonate (SALG 1%/SBC 1%); non-active coating with 1% sodium alginate (SALG 1%); active coating with 1.5% sodium alginate and 1% sodium bicarbonate (SALG 1.5%/SBC 1%); non-active coating with 1.5% sodium alginate (SALG 1.5%); active coating with 2% sodium alginate and 1% sodium bicarbonate (SALG 2%/SBC 1%); non-active coating with 2% sodium alginate (SALG 2%). Data are the average of three replicates ± standard error (SEM). Bars with different letters are significantly different (*p* < 0.05).

**Figure 4:**
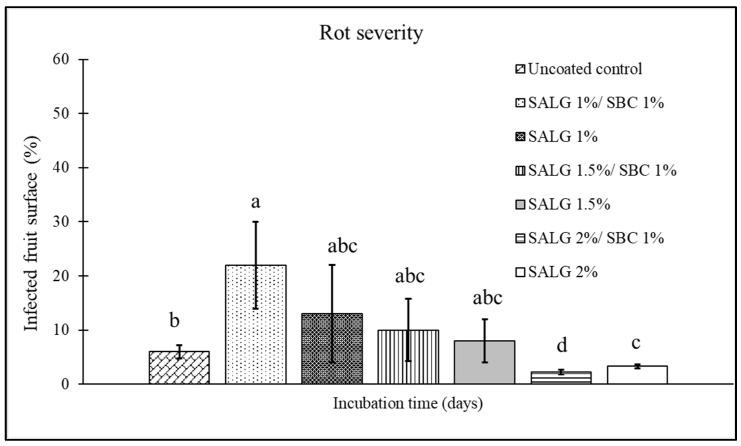
Disease severity (surface area of the infected fruit, %) on cherry tomatoes at 7 days of storage for the different treatments: control (uncoated); active coating with 1% sodium alginate and 1% sodium bicarbonate (SALG 1%/SBC 1%); non-active coating with 1% sodium alginate (SALG 1%); active coating with 1.5% sodium alginate and 1% sodium bicarbonate (SALG 1.5%/SBC 1%); non-active coating with 1.5% sodium alginate (SALG 1.5%); active coating with 2% sodium alginate and 1% sodium bicarbonate (SALG 2%/SBC 1%); non-active coating with 2% sodium alginate (SALG 2%). Data are the average of three replicates ± standard error (SEM). Bars with different letters are significantly different (*p* < 0.05).

The effect of incorporating 1% sodium bicarbonate into sodium alginate solution, at the concentrations (2, 1.5, and 1%) selected in Section 3.1, was tested in terms of the prolonged shelf-life of coated tomatoes. Specifically, a comparison was made between the two types of coating: non-active (sodium alginate and calcium chloride) and active (sodium alginate + sodium bicarbonate and calcium chloride). The incidence (Figure 3) and the severity (Figure 4) of the disease were evaluated in artificially inoculated tomatoes. As compared to an average incidence of 30% of infection in uncoated fruits, the non-active coatings, independent of the sodium alginate percentage, failed in reducing the incidence of rots (Figure 3). Instead, the addition of 1% sodium bicarbonate to 2% sodium alginate reduced disease incidence by 53%, as compared to the uncoated control, and by 44% as compared to its non-active coating counterpart.

Concerning disease severity (Figure 4), fruits coated with 1 and 1.5% alginate showed values comparable to the control, whereas, for the coating formulated with 2% sodium alginate, the active treatment (+1% SBC) produced a 67% decrease in severity as compared to uncoated fruit. This value resulted in higher than that recorded for the non-active coating (SALG 2%), which showed a 50% reduction in severity as compared to the control.

Sodium bicarbonate is a strong electrolyte that, upon contact with water, releases bicarbonate ions (HCO_3_^−^); the latter, in turn, behaves as a weak base that could affect the pH of the coating solution. A higher or more precisely controlled pH can enhance the formation of bonds between alginate and Ca^2+^ ions, thereby improving cross-linking and subsequently strengthening gel formation [22]. Furthermore, the presence of sodium bicarbonate could delay a prompt precipitation of the gel, giving enough time for a more even distribution of alginate and Ca^2+^ and thus a more homogeneous coating, reducing defects, porosity, and microcracks. Indeed, a well-crosslinked alginate gel (i.e., with a higher number of Ca^2+^- alginate bridges) tends to have better mechanical properties (greater hardness, less deformation), less solubility (less breakdown in water), and potentially better oxygen and moisture barrier properties [23]. However, this outcome is feasible only when the bicarbonate concentration is compatible with the system, ensuring that Ca^2+^ release is not hindered by CaCO_3_ formation or other unwanted precipitation. The combination of sodium bicarbonate/sodium alginate at 1:2 seems to fulfil those requirements.

### 3.3. Effect of Moringa Leaf Extract (MLE) on the Growth of Postharvest Fungi

**Figure 5:**
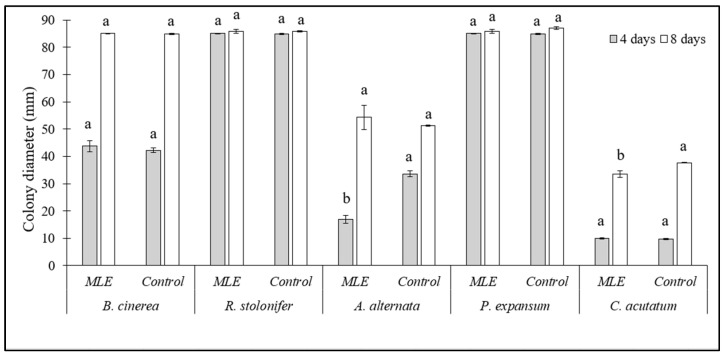
Colony growth (mm) of *Botrytis cinerea*, *Alternaria alternata*, *Rhizopus stolonifer*, *Colletotrichum acutatum*, and *Penicillium expansum* on PDA amended with 1.5% *Moringa* leaf extract (MLE). Unamended PDA plates were used as controls. Data are the average of three replicates ± standard error (SEM). For each pathogen and time point (4 and 8 days of incubation), bars with different letters are significantly different (*p* < 0.05).

The effect of 1.5% MLE on the growth of a selection of fungi known to be involved in the postharvest spoilage of the products under investigation is reported in Figure 5. Except for *A. alternata* at 4 days of incubation (−44%) and *C. acutatum* at 10 days of incubation (−10%), MLE did not show any effect on colony growth of the tested fungi as compared to their respective controls. Therefore, the putative effect on rots in *in vivo* assays may not result from a direct effect on the pathogen, as already suggested by Admane et al. [18], but from the presence in the extract of bioactive molecules as neochlorogenic acid, quercetin 3-O-β-d-glucopyranoside, glucomoringin, acetyl glucomoringin, caffeic acid, coumaroylquinic acid, and kaempferol diglucoside, with a role in the induction of resistance to stresses [24]. Whereas the antifungal activity of 1% sodium bicarbonate against the same fungi tested herein has been reported in a previous investigation [15], in which the salt resulted in effective, albeit to a lower degree, inhibition of *A. alternata* and *C. acutatum*, whose growth was reduced by no more than 50% even at the highest tested concentration (10 g/L).

### 3.4. Effect of the Active Edible Coating in Reducing Spoilage

**Figure 6:**
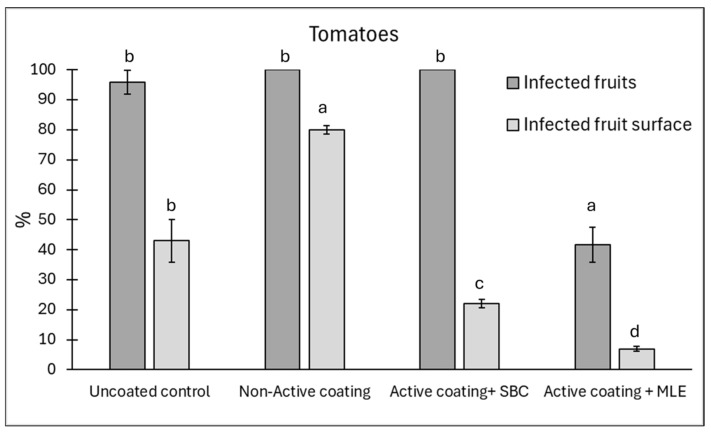
Disease incidence (infected fruits, %) and severity (infected fruit surface, %) at 12 days of storage on cherry tomato fruit wounded and inoculated with *Alternaria alternata*. Tomatoes were coated with an active coating, added with sodium bicarbonate (SBC) or *Moringa* Leaf Extract (MLE), or a non-active coating. Uncoated tomatoes served as a control. Data are the average of three replicates ± standard error (SEM). For each parameter, bars with different letters are significantly different (*p* < 0.05).

**Figure 7:**
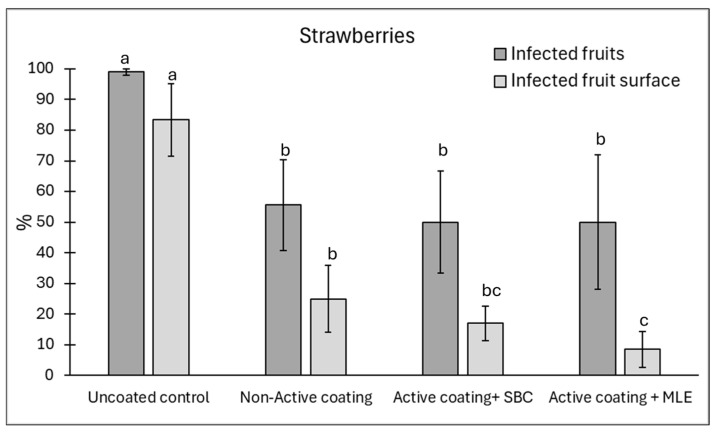
Disease incidence (infected fruits, %) and severity (infected fruit surface, %) on strawberries at 12 days of storage. Natural infections were evaluated. Strawberries were coated with active, added with sodium bicarbonate (SBC) or *Moringa* Leaf Extract (MLE), or non-active. Uncoated strawberries served as a control. Data are the average of three replicates ± standard error (SEM). For each parameter, bars with different letters are significantly different (*p* < 0.05).

**Figure 8:**
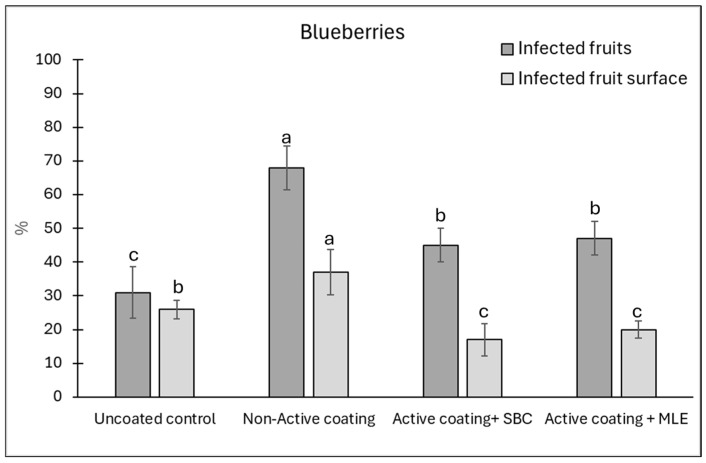
Disease incidence (infected wounds, %) and severity (infected fruit surface, %) at 12 days of storage on blueberries wounded and inoculated with *Botrytis cinerea*. Blueberries were coated with active, added with sodium bicarbonate (SBC) or *Moringa* Leaf Extract (MLE), or a non-active coating. Uncoated blueberries served as a control. Data are the average of three replicates ± standard error (SEM). For each parameter, bars with different letters are significantly different (*p* < 0.05).

Following the results reported in Section 3.2, an assay with active edible coating was conducted on different commodities: tomato (Figure 6), strawberries (Figure 7), and blueberries (Figure 8). The active ingredients were 1% sodium bicarbonate and 1.5% MLE. Overall, the MLE-containing coating was the most effective in reducing the incidence of postharvest rots (Supplementary Figure S2), particularly on tomatoes artificially inoculated with *A. alternata* (Figure 6). On this latter, disease incidence and severity were reduced by 59 and 73%, respectively, whereas sodium bicarbonate did not affect disease incidence but it reduced disease severity by 53%. In the presence of a non-active coating, a higher severity was recorded compared to uncoated control samples, likely due to pathogen conidia present on the fruit surface, which the coating maintained under favourable humidity. This finding seems to support the ability of the coating to reduce the transpiration, consequently preventing the weight loss, which is a relevant threat to the product’s shelf-life. As such, the use of the active coating might prolong the storability, reducing both weight loss and rots. This finding is consistent with the fungistatic effect observed in vitro on *A. alternata*.

In strawberry fruits, the addition of MLE and sodium bicarbonate did not improve the coating’s efficacy against disease incidence, which was reduced on average by 45% (Figure 7). However, active coating containing MLE significantly reduced disease severity by 88% as compared to the uncoated control.

The blueberries exhibited a different trend, with the coating showing no reduction in the disease incidence as compared to the control (Figure 8). However, as observed on tomatoes, the inactive coating increased both disease incidence and severity. However, the inclusion of active agents, especially sodium bicarbonate, seemed to lower the incidence as compared to the non-active coating, reaching values similar to those of the uncoated control. A better reducing effect was recorded on disease severity, which was reduced by 43 and 29% in the presence of sodium bicarbonate and MLE active coatings, respectively, as compared to the uncoated control, and by 58 and 47%, respectively, as compared to the non-active coating samples. These results seem in agreement with the antifungal effect of 1% sodium bicarbonate against *B. cinerea* recorded in a previous investigation [15].

The differing behaviours observed among the commodities may be attributed to their distinct intrinsic characteristics. For example, the texture of the fruit surface might play a critical role in the efficacy of edible coatings. Rough or porous surfaces (e.g., strawberries) can trap air bubbles or coating material. Smooth surfaces (e.g., tomatoes, blueberries) might allow for more uniform film formation, improving the coating’s protective function. Furthermore, the hydrophobicity/hydrophilicity of the fruit’s surface might affect the coating spreading: hydrophobic surfaces (e.g., citrus natural wax) might repel water-based coatings, leading to poor adhesion. Hydrophilic surfaces can promote better coating spread and adhesion but may also absorb moisture, weakening water-based films.

Concerning the MLE active coating, a role could have been played by the high phytochemical constituents in *M. oleifera* extract, including phenols, alkaloids, and tannin compounds, which exert interesting antioxidant and antifungal potential as reported by Anyasor et al. [25], Kubheka et al. [26], and Admane et al. [18]. Similarly, Garcia and Davidov-Pardo [27] reported that lipophilic compounds in *Moringa* extracts are known to efficiently bind to fungal cytoplasmic membranes, increasing permeability and retarding growth. This could be corroborated by the findings of Maqbool et al. [28] and Ali et al. [29], who reported that the incorporation of plant-based active ingredients, such as essential oils and plant extracts, might significantly improve the antimicrobial activity of edible coatings against postharvest pathogens that cause major economic losses.

## 4. Conclusions

The use of edible coatings proved to be effective in reducing the severity of rots on naturally infected cherry tomatoes. In particular, the best performance was achieved with a first dip in a combination of 2% sodium alginate and 1% sodium bicarbonate, followed by 3 min dipping in 2% calcium chloride. In this case, both the action of sodium alginate, which creates a thin physical barrier around the tomato, and the presence of sodium bicarbonate, a salt with known antimicrobial properties and used as a food preservative, reduced the incidence/severity of rots in treated tomatoes. A further test using higher pathogen inoculum pressure was conducted with active edible coatings made up with sodium bicarbonate or *M. oleifera* leaf extract on different host–pathogen combinations, suggesting that the active edible coatings could reduce the rots, thus contributing to prolong the shelf-life of cherry tomato, blueberry, and strawberry fruits as compared to the uncoated control or the non-active coating. These edible coatings could therefore be an alternative organic postharvest treatment to be used by the agrifood industries. Furthermore, given the high content of bioactive principles, the use of *M. oleifera* in edible coatings might also add functionality to the coated foods.

## Figures and Tables

**Table 1:** Combinations of different concentrations (*w*/*v*) of sodium alginate and calcium chloride used in the preparation of the coating and of different immersion times (min) in calcium chloride.

Concentration (*w*/*v*)		Immersion in Calcium Chloride Solution (min)
Sodium Alginate Solution	Calcium Chloride Solution	
2%	2%	3
		6
	4%	3
		6
1.5%	2%	3
		6
	4%	3
		6
1%	2%	3
		6
	4%	3
		6

**Table 2:** Description of the combinations of sodium alginate, calcium chloride, and sodium bicarbonate used in the preparation of the coating and of the immersion times in calcium chloride.

Concentration (*w*/*v*)			Immersion in Calcium Chloride (min)
Sodium Alginate	Calcium Chloride	Sodium Bicarbonate	
2%	2%	0%	3
		1%	
1.5%	2%	0%	6
		1%	
1%	2%	0%	3
		1%	

## Data Availability

The original contributions presented in the study are included in the article/Supplementary Material, further inquiries can be directed to the corresponding author.

## Supplementary Materials

The following supporting information can be downloaded at: https://www.mdpi.com/article/10.3390/foods15010011/s1, Figure S1: Protocol for active/non active coating preparation; Figure S2: Active/non active coatings of different fruits.

## Abbreviations

MLE	*Moringa oleifera* leaf extract
EFSA	European Food Safety Authority
GRAS	Generally Recognised as Safe
FDA	United States Food and Drug Administration
PDA	Potato Dextrose Agar
SEM	Standard error of the mean
SBC	Sodium bicarbonate
SALG	Sodium alginate
