# Supplementary material for: Active Edible Coatings to Mitigate Postharvest Diseases Causing Waste of Blueberries, Strawberries, and Cherry Tomatoes
Source: Foods. 2025 Dec 19;15(1):11. doi: 10.3390/foods15010011 (PMC12785490; doi:10.3390/foods15010011)
Supplement: Supplementary file 1 [file foods-15-00011-s001.zip › Supplementary figure S1.pdf]

NON-ACTIVE COATING

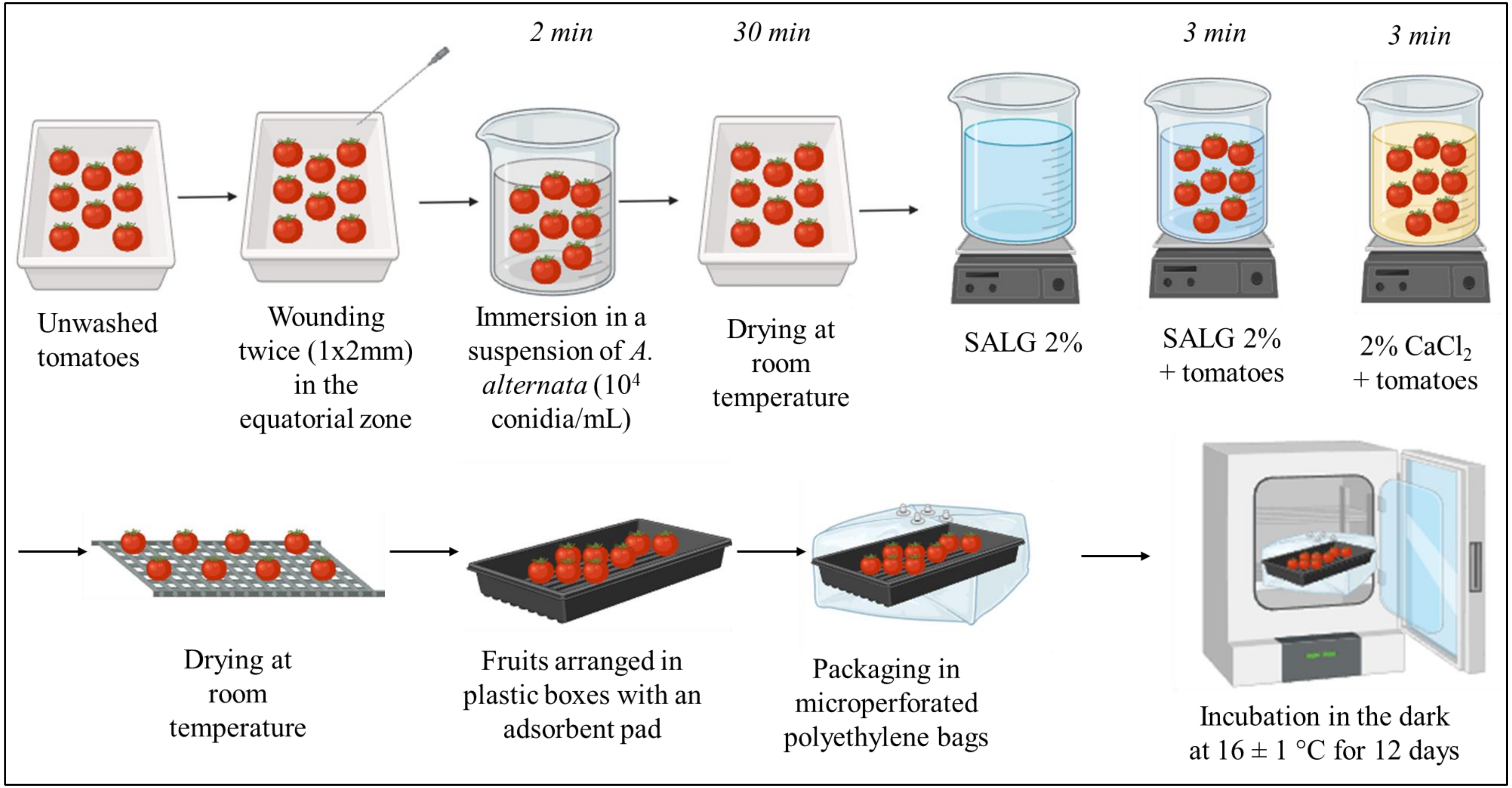

Workflow of the non-active coating process applied to tomatoes: immersion in 2% sodium alginate (SALG) followed by 2% calcium chloride ($CaCl_2$) solutions, drying, arrangement in plastic boxes with an adsorbent pad, packaging, and storage in an incubator.

Created in https://BioRender.com

ACTIVE COATING

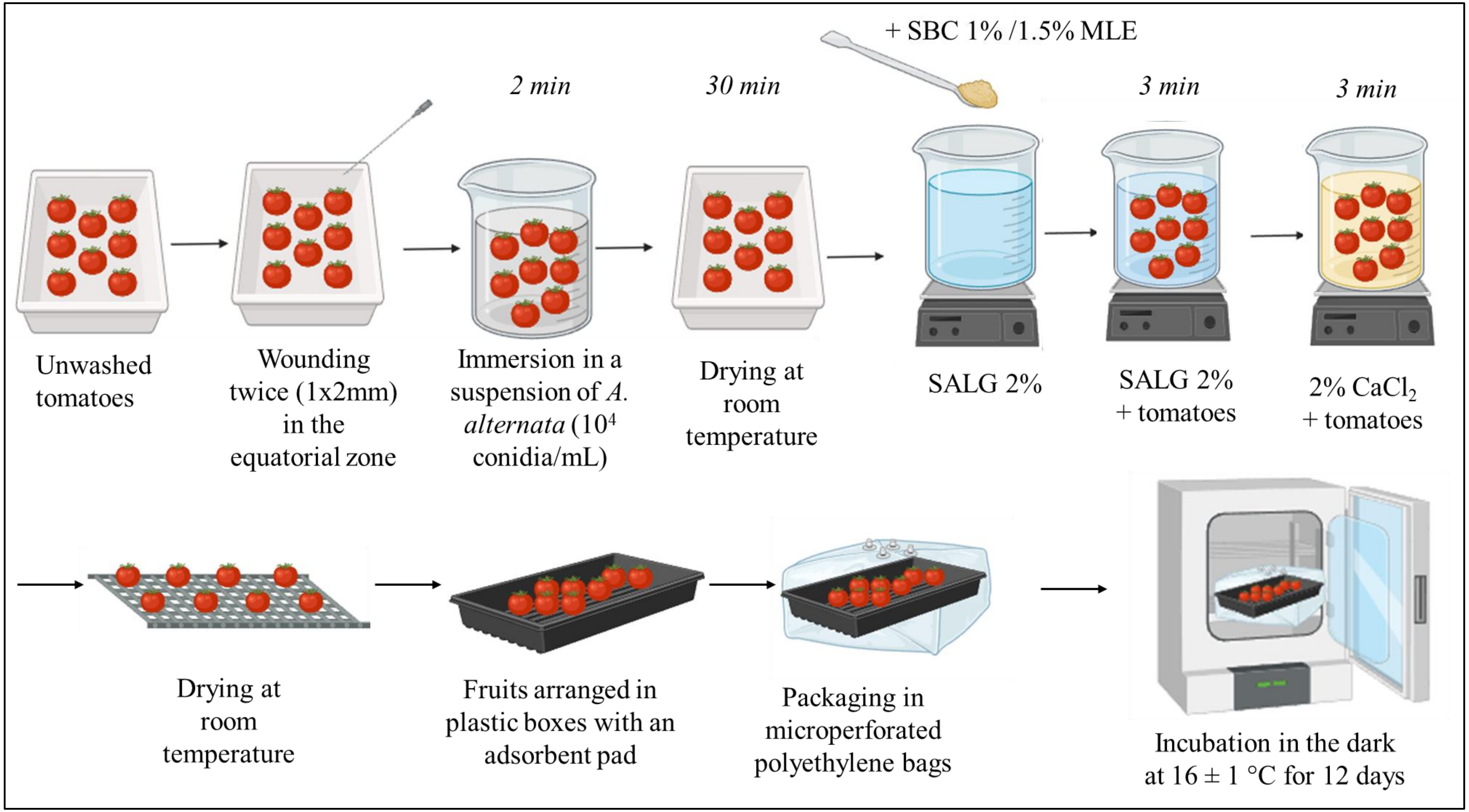

Workflow of the active coating process applied to tomatoes: including immersion in 2% sodium alginate (SALG) amended with 1% sodium bicarbonate (SBC) or 1.5% *Moringa* leaf extract (MLE), followed by 2% calcium chloride ($CaCl_2$) solutions, drying, arrangement in plastic boxes with an adsorbent pad, packaging, and storage in an incubator.

Created in https://BioRender.com
