# Supplementary material for: Active Edible Coatings to Mitigate Postharvest Diseases Causing Waste of Blueberries, Strawberries, and Cherry Tomatoes
Source: Foods. 2025 Dec 19;15(1):11. doi: 10.3390/foods15010011 (PMC12785490; doi:10.3390/foods15010011)

Strawberries
12 days from edible coating application
Storage temperature: 5°C

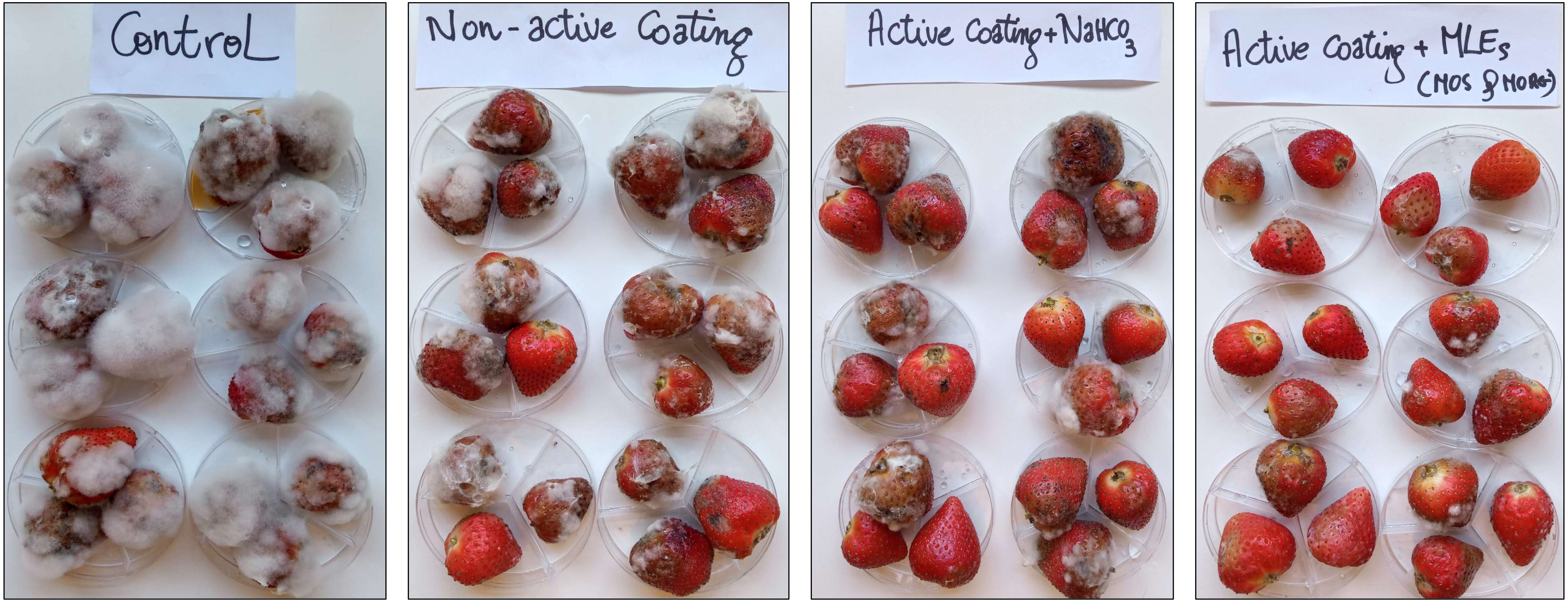

Cherry tomatoes
12 days from edible coating application
Storage temperature: 16 °C

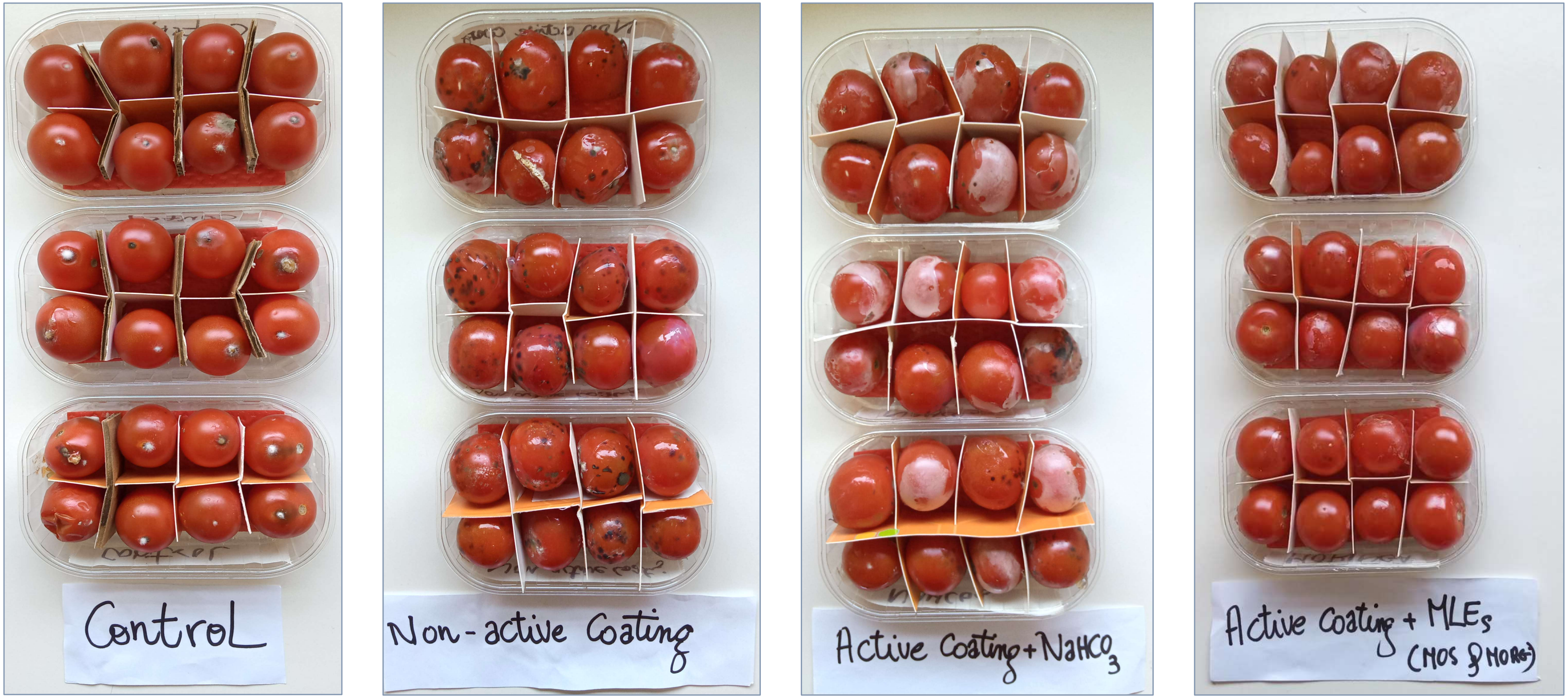

Blueberries
12 days from edible coating application
Storage temperature: 16 °C

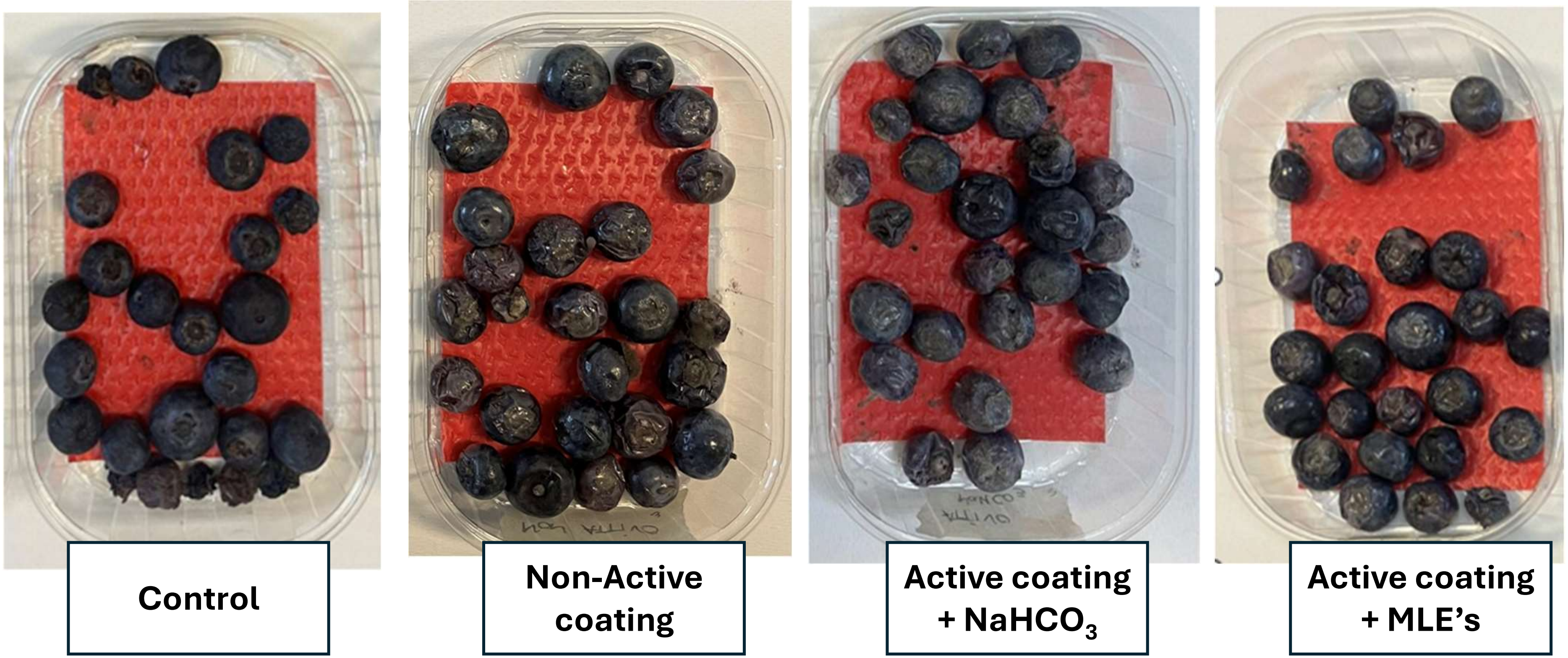

Supplement: Supplementary file 1 [file foods-15-00011-s001.zip › Supplementary figure S2.pdf]
